# Non-thoracic Source of Bleeding During Left-sided Thoracic Surgery

**DOI:** 10.7759/cureus.4593

**Published:** 2019-05-03

**Authors:** Jonathan B Cohen, Michael R Hirschi, Sephalie Y Patel, Jinhong Liu

**Affiliations:** 1 Anesthesiology, H. Lee Moffitt Cancer Center and Research Institute, Tampa, USA; 2 Anesthesiology, University of South Florida, Tampa, USA

**Keywords:** anesthesia, thoracic surgery, bleeding

## Abstract

Hypotension during thoracic surgery is traditionally attributed to intrathoracic causes such as pulmonary bleeding, ventilation, causing decreased venous return, and a decrease in myocardial contractility. We present a case of unexplained hypotension presenting at the end of left-sided thoracic surgery. The cause of hypotension was ultimately found to be due to intra-abdominal bleeding from a splenic injury. This case reminds the anesthesiologist to be vigilant of non-thoracic causes of hypotension during left-sided lung surgery.

## Introduction

Crisis management during anesthesia involves the review and treatment of probable causes. Hypotension is one of the most common aberrancies that an anesthesiologist may observe during general anesthesia, affecting between 5% and 99% of patients, depending on the definition used [1-2]. Nonetheless, its presence must be met with an appropriate degree of concern. In elderly patients, even modest hypotension may result in the underperfusion of vital organs [1,3]. In a French survey, intraoperative hypotension was associated with anesthetic-related death [4]. Acute, severe hypotension may herald or precipitate cardiac arrest [1].

Establishing the correct etiology of a cause of the patient’s hypotension is essential. Complicating the diagnostic process is that causes of hypotension during anesthesia are myriad and often multifactorial [5-6]. Conceptualizing the circulation as a simple hydraulic model consisting of three parts (intravascular volume (fluid), cardiac contractility (pump), and vascular tone (pipes)) may assist the anesthesiologist with identifying possible causes of hypotension.

When hypovolemia due to hemorrhage is suspected, how should the team proceed when no bleeding is identified by the surgeon despite a decreasing hemoglobin concentration? During thoracic surgery, intra-abdominal sources of bleeding should be considered, as the diaphragmatic border may be compromised.

## Case presentation

The patient was an 82-year-old male who was scheduled for a robotic left lower lobectomy for the resection of a biopsy-proven squamous cell lung carcinoma. He had a medical history of hypertension controlled with multiple medications; a 40-pack-year history of tobacco abuse, with a 20-year history of abstinence; and a prior history of melanoma of his left upper extremity, which was successfully resected.

**Figure 1:**
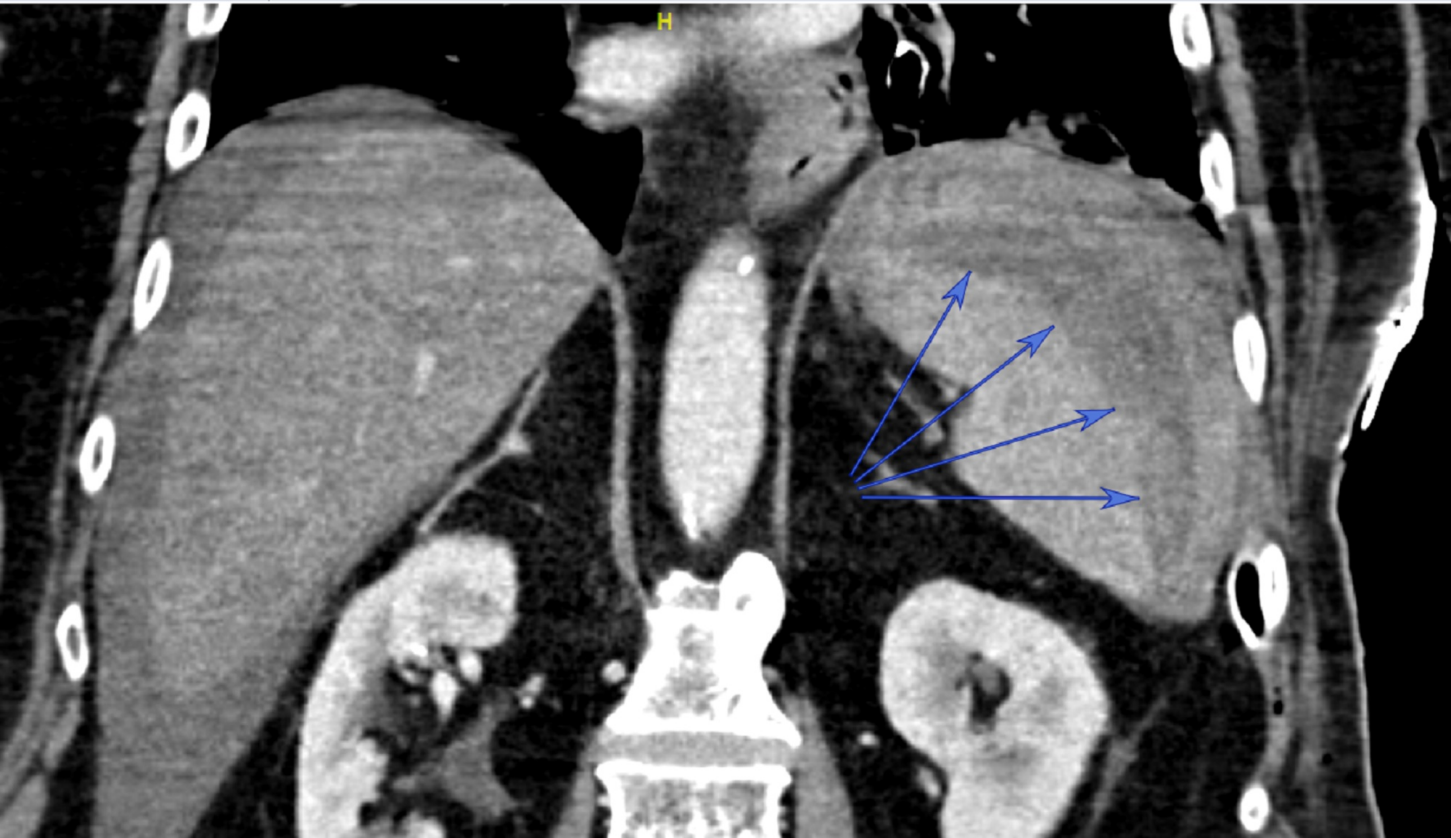
Coronal view from computed tomography scan Blue arrows identify splenic hematoma

Standard American Society of Anesthesiology (ASA) monitors were placed and preoxygenation was performed; general anesthesia was induced with propofol, followed by the administration of muscle relaxant and the placement of a left-sided, double-lumen endotracheal tube. Additional venous access and arterial line placement occurred concurrently while the correct placement of the endotracheal tube was confirmed by bronchoscopy. The placement was then reconfirmed after the patient was situated in the right lateral decubitus position. The left lung was isolated and deflated, and the surgeon proceeded to place ports to facilitate the robotic resection as follows: the sixth intercostal space in the anterior axillary line, the third intercostal space in the anterior axillary line, and the ninth intercostal space in the posterior axillary line. The surgical procedure continued uneventfully from an anesthesia perspective with level 7 and 10 lymph node dissections, extensive lysis of adhesions and pericardial and pleural fat to facilitate visualization, and a left lower lobectomy. Surgical blood loss was estimated at less than 50 cc. Chest tubes were placed through the sixth and ninth intercostal port incisions. A bolus of 30 cc of 0.5% bupivacaine was administered via a catheter placed at the tenth intercostal space in the posterior axillary line for an elastomeric pump ball to infuse local anesthesia for post-operative pain management. Within 15 minutes of this, the patient began to experience hypotension, requiring incrementally increasing doses of vasopressors. Over the course of 10 minutes, escalating doses of phenylephrine were becoming ineffective at restoring the patient’s mean arterial pressures (MAP) to his intraoperative baseline (within 20% of the patient’s preoperative MAP). Vasopressin was administered, in 1-2 mg aliquots, as urgent assistance was summoned to the operating room. The hemodynamic instability was immediately reported to the surgeon. Based on the scant chest tube drainage, a thoracic source of bleeding was considered unlikely. A portable chest radiograph was ordered and point-of-care blood testing was performed. The hemoglobin was resulted at 6.5 g/dL, and transfusion was initiated while awaiting a confirmatory hemoglobin sent to the lab, which was resulted at 7.4 g/dL (preoperative hemoglobin 13. 5 g/dL). The chest radiograph did not show any areas suspicious for bleeding. The double-lumen endotracheal tube was removed and replaced with a single-lumen endotracheal tube to facilitate transport and the potential for prolonged mechanical ventilation, and the patient was transported urgently to the computed tomography (CT) scanner for chest, abdomen, and pelvis imaging. Upon review with the radiologist, the diagnosis was made of a subcapsular splenic hematoma with fluid extending to the diaphragm and tracking along the paracolic gutters, concerning for a splenic injury (Figure 1).

The patient was then returned to the recovery room, where he continued to require ongoing blood transfusion and phenylephrine infusion to maintain satisfactory blood pressures. After receiving a total of four units of packed red blood cells, the phenylephrine infusion was able to be weaned significantly and serial hemoglobin values stabilized. Although both splenectomy and splenic embolization were considered in this patient, as his hemodynamic instability abated, the decision was made to observe him in the intensive care unit and to continue serial hemoglobin monitoring. On the first postoperative day (POD), he was weaned completely off of phenylephrine and was extubated. He was discharged to a rehabilitation facility on POD 15.

## Discussion

In this case, there was a high suspicion of hemorrhage-related hypotension due to no apparent signs of a decrease in cardiac contractility (no obvious electrocardiogram changes consistent with ischemia or infarction) or suggestion of vasodilation (no concurrent signs of anaphylaxis). This was confirmed by the precipitous drop in hemoglobin on point-of-care testing. Further imaging disclosed the origin of the bleeding was from a location unexpected by the anesthesia and surgical team.

Splenic injury has been reported several times following left-sided thoracotomies [7-9]. Table 1 compares three earlier published cases with our own. In all four cases, the procedure was performed on the left side. In contrast, as our patient was under general anesthesia, there was no episode of coughing that could have contributed to splenic laceration, nor was CPR performed. Additionally, our patient was managed conservatively without intervention for his splenic injury. It is very unlikely that the robotic-assisted laparoscopic technique used played any direct role in the splenic injury. The contemporaneousness of the bolus of bupivacaine and the onset of hypotension led to the initial incorrect assertion that it was the cause of the decrease in blood pressure, which became apparent as the hypotension continued to progress despite treatment.

**Table 1:** Summary of reports of splenic injury during thoracotomy

Case	Operation	Time to presentation	How diagnosis made	Findings	Outcome	Postulated Cause
Klotz et al. [7]	Left thoracoscopy converted to thoracotomy for L inferior lobe resection	15 hours postop	Abdominal ultrasound	4L hemoperitoneum, bleeding from rupture of encapsulated hematoma from the upper pole of the spleen. Total splenectomy performed. Upper abdominal adhesions noted.	Discharged home, POD #12	Spontaneous rupture vs. mobilization of adhesions between diaphragm and spleen
Acharya et al. [8]	Left thoracotomy for left upper lobe sleeve resection converted to left pneumonectomy	Excessive chest tube drainage & cardiac arrest leading to re-operation and satisfactory repair of suture line & hemostasis, but unable to resuscitate patient	Autopsy	1.2 L hemoperitoneum with subcapsular hematoma at the upper pole and lacerations at the upper and lower poles. Multiple (2-7^th^) left-sided rib fractures.	Expired	Elevation of diaphragm into the left chest after pneumonectomy caused the spleen to reside higher anatomically and predisposed it to injury during chest compressions initiated during cardiac arrest
Stupnik et al. [9]	Left thoracotomy for left lower lobectomy and sleeve resection of left upper lobe bronchus	POD # 3	Abdominal ultrasound	Large hematoma around the spleen with lacerations at the upper pole extending into the parenchyma. Total splenectomy performed. No adhesions identified in the abdomen.	Discharged home, POD # 20	Violent coughing episode on POD # 3
Our case	Robotic left lower lobectomy	Intraoperatively	CT scan	Subcapsular splenic hematoma with fluid extending to the diaphragm and tracking along the paracolic gutters	Discharged home POD # 15	

As summarized in Table 1, splenic injury has been reported several times following left-sided thoracotomies [7-9]. In the first case, the patient developed signs of shock 15 hours after a left-sided thoracotomy for wedge resections of nodular tumors [7]. The diagnosis was made after an ultrasound of the abdomen was performed after a blood panel demonstrated hemoglobin of 5 g/dL. On laparotomy, the rupture of an encapsulated splenic hematoma resulted in a large (4L) hemoperitoneum, necessitating splenectomy. The authors opined in this case that this was a likely case of spontaneous splenic rupture after ruling out idiopathic injury and evidence of pathological processes associated with a heightened risk of splenic rupture. They considered that the mobilization of adhesions between the diaphragm and the spleen during intraoperative thoracic manipulation to also be a possibility. In the second case, a tumor involving the orifice of the left upper lobe bronchus required a pneumonectomy when lung re-inflation was not achieved after a sleeve resection [8]. The patient suffered cardiac arrest immediately postoperatively, requiring cardiopulmonary resuscitation and emergent thoracotomy for the repair of a disrupted pulmonary vein suture line. The patient subsequently expired, and splenic injury was observed on postmortem examination. In this case, the injury was believed to be a consequence of cardiopulmonary resuscitation. In the last case, a thoracotomy for left lower lobectomy with sleeve resection of the left upper lobe bronchus was performed for metastatic vulvar cancer [9]. On postoperative day 3, she developed signs of hemorrhage shock, the source of which was confirmed with an abdominal ultrasound. The injury was believed, in this case, to be caused by a severe bout of coughing, resulting in splenic rupture.

Two of the case reports allude to the notion that left-sided lung resection can elevate the diaphragm and apply tension to the splenic hilum, contributing to its rupture [8-10]. Two cases also reference the role that adhesions from prior abdominal operations (cholecystectomies in both) may play in anchoring the spleen [7-8]. Of note, our patient also had a prior cholecystectomy. Although four case reports lack the power to suggest the definitive causality of splenic injury after thoracotomy, some interesting observations can be made. All four cases involved left-sided operations, and three of the four cases occurred in patients who had previously had cholecystectomies. It is possible that the elevation of, or tension on, the diaphragm after left lung resection, in concert with abdominal adhesions, predisposes the spleen to injury.

## Conclusions

Crisis management during anesthesia requires vigilance and investigation into abnormal vital signs and laboratory values. Regardless of the exact mechanism of injury, these cases serve as a reminder to the anesthesiologist to consider splenic injury in cases of shock during and after left-sided lung surgery.
